# Expression of matrix metalloproteinases-12 in ST-segment elevation myocardial infarction: A case–control study

**DOI:** 10.1097/MD.0000000000008035

**Published:** 2017-10-27

**Authors:** Jing Wang, Guoqing Wei, Wei Hu, Linhua Li, Yujia Ye, Huawei Wang, Wen Wan, Rui Li, Longjun Li, Linling Ma, Zhaohui Meng

**Affiliations:** Laboratory of Molecular Cardiology, Department of Cardiology, The First Affiliated Hospital of Kunming Medical University, Kunming, P.R. China.

**Keywords:** coronary thrombus, MMP12, STEMI, TIMP1

## Abstract

Matrix metalloproteinases-12 (MMP12) can lead to degradation of elastin resulting in plaque destabilization and rupture. MMP12 also facilitates platelet aggregation, adhesion, and granule secretion. However, evidence in the literature related to the function of MMP12 in ST-segment elevation myocardial infarction (STEMI) is little. This study investigated the expression of MMP12 in human coronary thrombus and examined the relationship between plasma MMP12 and STEMI.

Arterial plasma was obtained from 46 STEMI patients and 52 stable angina pectoris (SAP) patients and 30 controls with angiographically normal coronary arteries. Coronary thrombi were obtained from 26 STEMI patients with a large thrombus burden (LTB). The expression levels of MMP12 in coronary thrombus were analyzed by immunohistochemistry and immunofluorescence, reverse transcription-polymerase chain reaction (RT-PCR), Western blotting (WB) and casein zymography. In addition, MMP12 concentration measured by enzyme-linked immunosorbent assay (ELISA) and activity measured by fluorescence resonance energy transfer (FRET) were used to assess the levels in plasma.

We confirmed the expression of MMP12 in human coronary thrombus. MMP12 was secreted mainly in active form of 45 kDa in coronary thrombus. In plasma samples of the STEMI group, MMP12 concentrations were found to be higher than the SAP group (5.030 ± 2.24 pg/mL vs 3.010 ± 1.99 pg/mL, *P* < .05) but with lower MMP12 activity (332 ± 77 RFU vs 458 ± 91 RFU, *P* < .05). Also, the STEMI group demonstrated much higher MMP12 concentrations than the normal coronary artery control group (5.030 ± 2.24 pg/mL vs 1.720 ± 0.51 pg/mL, *P* < .05) and with lower MMP12 activity (332 ± 77 RFU vs 549 ± 112 RFU, *P* < .05). In addition, the STEMI group had significantly higher tissue inhibitor of metalloproteinases-1 (TIMP1) concentration (573.40 ± 270.60 pg/mL) than SAP group (384.50 ± 147.70 pg/mL) and control group (219.90 ± 154.80 pg/mL, *P* < .05). The imbalance in MMP12/TIMP ratio was observed in the STEMI group compared with SAP and control group (*P* < .05).

This study demonstrated that MMP12 exists in human coronary thrombus. Patients with STEMI have elevated plasma level of MMP12 and the imbalance of MMP12/TIMP1. These data supported that MMP12 might be of potential relevance in STEMI.

## 1 Introduction

Around the world, coronary artery disease (CAD) is the leading cause of death, where over 7 million people die from CAD every year, accounting for 12.8% of all deaths.^[1]^ Approximately 1 out of every 6 men and 1 out of every 7 women will die from myocardial infarction, averaging 1 death every 40 seconds.^[1]^ Acute myocardial infarction is primarily initiated by thrombosis caused by plaque rupture or erosion.^[2]^ Over the past 4 decades, percutaneous coronary intervention (PCI) has become a cornerstone for the treatment of myocardial infarction patients. Although PCI has increasingly targeted complex lesions and enhanced the treatment of urgent cases with a remarkably high success rate, the thrombus-containing plaque remains a considerable revascularization challenge.^[3]^ The thrombus burden (TB) is an independent predictor of increased myocardial damage, stent thrombosis, in-hospital complications, recurrent MI, and rates of mortality.^[4]^ Platelets play a key role in the formation and extension of atherosclerotic plaques,^[5]^ and the membrane proteins expressed on their surface are critical for the aggregation, adhesion, and release.

The MMP family is comprised of 25 related, but distinct, vertebrate gene products that play roles in many physiological processes and pathological processes, such as immunity, bone remodeling, inflammation and atherosclerosis.^[6]^ MMPs have recently emerged as important mediators of platelet function and thus thrombotic diseases. The high expression of MMPs in the margin and shoulder of plaques induced by coronary atherosclerosis can degrade more extracellular substrate than is being synthesized, thus leading to rupture of the fiber cap. Previous findings demonstrated that MMP1, MMP2, MMP8, MMP9, and MMP14 are present in thrombectomy samples obtained from patients with acute myocardial infarction.^[7]^

Among MMPs, macrophage metalloelastase also known as matrix metalloproteinase 12 (MMP12) is a 54 kDa proenzyme that is processed into a 45 kDa and then a 22 kDa active forms. MMP12 can lead to degradation of elastin,^[8]^ resulting in increased elastin fragment that plays a key role in plaque destabilization. In addition, our group recently reported that human platelets express MMP12 and MMP12 can facilitate type I collagen induced platelet aggregation, adhesion, and alpha granule secretion by cleaving the CEACAM1 exodomain on platelet membrane.^[9]^ Based on these findings, we raised the hypothesis that MMP12 is involved in coronary thrombogenesis and is potential relevance in STEMI. To the authors’ knowledge, this present study is the first report of MMP12 expression in coronary thrombi. In addition, elevated plasma concentrations of MMP12 and the imbalance of MMP12/TIMP1 ratio might be related with STEMI. Targeting this pathway with therapeutics is a promising strategy for treatment of patients with STEMI.

## 2 Methods

### 2.1 Study population

We used a case–control design with a total of 128 subjects recruited from the First Affiliated Hospital of Kunming Medical University during from December 2014 to December 2016. The study was comprised of three patient groups. Group I consisted of patients with ST-segment elevation myocardial infarction (STEMI, n = 46) undergoing primary PCI. STEMI was defined as a rise in troponin values above the 99th percentile of the upper reference limit in conjunction with at least one of the following: symptoms of ischemia, new ST-segment elevation, or left bundle-branch block. Within group I, we used a thrombus grading classification system based on visual angiographic assessment of the thrombus size using a score ranging from grade 0 to 5.^[10]^ Within the STEMI group, the large thrombus burden (LTB, n = 26) subgroup was defined as those with thrombus grade 4 or 5, while the small thrombus burden (STB, n = 20) subgroup had thrombus grades of 1 to 3. Group II was comprised of patients with stable angina pectoris (SAP, n = 52) undergoing coronary angiography. Group III (controls, CON, n = 30) included patients with atypical chest pain undergoing diagnostic coronary angiography with angiographically normal coronary arteries (defined as having a smooth luminal surface in the absence of stenosis). Exclusion criteria included the need for emergency grafting of a coronary artery bypass and an inability to provide oral informed consent.

The protocol was approved by the Ethics Committee of Kunming Medical University and all patients gave written informed consent to participate in this study.

### 2.2 Experimental protocol

#### 2.2.1 Sample collection and storage

Blood samples (4.5 mL) were obtained from radial arteries by sheath and collected in prechilled Vacutainer tubes containing 0.5 mL of CTAD (a mixture of citrate, theophylline, adenosine, and dipyridamole) and stored in crushed ice. Blood samples were centrifuged at 1000 *g* at 4°C for 20 minutes and the supernatant plasma was stored in separate aliquots at –80°C.

In addition, only patients (n = 26) with STEMI-LTB underwent manual thrombus aspiration, because recently 2 large RCT reported that routine thrombus aspiration during PCI for STEMI did not reduce major adverse cardiovascular and cerebrovascular events (MACCEs).^[11,12]^ After the lesion was crossed with a guidewire, the thrombectomy device was advanced and suction started before it crossed the lesion. Following a wash with 0.9% normal saline, coronary thrombus samples were stored at –80°C. Samples pertaining to matched cases and controls were always analyzed together in the same batch and laboratory personnel were unable to distinguish among cases and controls.

#### 2.2.2 Assessment of MMP12 in thrombus

##### 2.2.2.1 Immunohistochemistry and immunofluorescence

Coronary artery thrombi were fixed in 10% phosphate-buffered formaldehyde immediately after thromboaspiration for 24 hours, embedded in paraffin and then sliced into serial sections of 4 μm thick. After incubation with a primary antibody: Anti-MMP12 antibody (EP1261Y, rabbit monoclonal antibody, corresponding human MMP12 aa E450 to the C terminal, MilliporeSigma, Darmstadt, Germany) overnight at 4°C, a secondary antibody (goat antirabbit IgG-B; Santa Cruz Biotechnology, Santa Cruz, CA) was incubated for 30 minutes at 37°C. Counterstaining was carried out with hematoxylin for visualization. Positive immunohistochemistry staining was in the form of yellowish brown or brown particles in the cytoplasm and cell stroma.

The coronary artery thrombus specimens were dipped in optimum cutting temperature compound (OCT) for 72 hours and then sliced into 6 μm frozen sections using a frozen slicer (LEICA CM1950). After fixing with acetone for 15 minutes (–20°C), the frozen sections were immersed in 1 × PBS (PH 7.4, 137 mM NaCl, 2.7 mM KCl, 10 mM Na_2_HPO_4_, 2 mM KH_2_PO_4_) for 10 minutes, blocked for 1 hour at 37°C in 1 × PBS containing 5% bovine plasma albumin and then incubated with MMP12 primary antibodies as previously for 2 hours at 37°C. After washing the sections, the primary antibody was detected by incubating with a fluorophore conjugated secondary antibodies to yield a green fluorescent product at the site of the antigen (Daylight 488 Conjugated donkey antirabbit IgG, 1:100 dilution; BETHYL, Montgomery, TX) for 2 hours at 37°C in the dark. Finally, the sections were incubated with 4′,6-diamidino-2-phenylindole (Invitrogen, Waltham, Massachusetts) for 5 minutes at RT in the dark to stain nuclei and visualized under a fluorescence microscope (Olympus BX51, Tokyo, Japan) after sealing with buffer glycerol. Meanwhile, images were acquired using CellSens standard-experiment documentation (Olympus). The negative control was incubated with 1 × PBS instead of primary antibody.

##### 2.2.2.2 Reverse transcription-polymerase chain reaction

To determine MMP12 mRNA levels, total RNA was isolated from coronary artery thrombus tissues (30 mg) after ground to a fine powder in liquid nitrogen by using the Trizol Reagent (1 mL) (Invitrogen). Approximately 0.5 μg of total RNA was reverse-transcribed to cDNA in a 20 μL volume using thermo scientific revert aid first strand cDNA synthesis kit K1621 (Thermo, Waltham, Massachusetts). Briefly, after incubation at 42°C for 1 hour, the reaction mixture was terminated by heating to70°C for 5 minutes. The synthesized cDNA was amplified by polymerase chain reaction (PCR).

Primers (GenScript, Nanjing, China) were designed to amplify MMP12 cDNA. The forward primer, 5′-CGATGAGGACGAATTCTGGACTAC-3′, is situated in exon 4, while the reverse primer, 5′-GGTTCTGAATTGTCAGGATTTGGC-3′, is situated in exon 6. The primer sequences correspond to residues D211 through P292 in the catalytic domain of human MMP12. Reaction conditions included an initial denaturation at 94°C for 2 minutes, followed by 35 cycles of denaturing at 94°C for 30 seconds, annealing at 50°C for 30 seconds, and extending at 72°C for 1 minute and a 10 minute final extension at 72°C. The quality of the total RNA was determined by RT-PCR for the house-keeping gene, glyceraldehyde-3-phosphate dehydrogenase (GAPDH).

##### 2.2.2.3 Western blotting

Thrombus tissues from STEMI-LTB patients were pulverized in liquid nitrogen. Samples (30 mg) were diluted in 200 μL 1 × PBS, centrifuged at 11,000 rpm for 15 minutes at 4°C and the supernatant was collected. Next, 3 μL of 5 × SDS–PAGE loading buffer was added to 15 μL supernatant, the sample was heated in boiled water for 15 minutes and then separated on 12% SDS–PAGE gels. The proteins were then transferred to polyvinylidene fluoride (PVDF) membranes. Recombinant human MMP12 (molecular weights of 45 kDa obtained from our laboratory as previously described^[13]^) was used as positive control. The membranes were subsequently immersed into anti-MMP12 antibody (EP1261Y, rabbit monoclonal antibody, corresponding human MMP12 aa E450 to the C terminal, MilliporeSigma) at a 1:1000 dilution for 2 hours at 37°C and goat antirabbit IgG-B (Santa Cruz Biotechnology) at a 1:1000 dilution for 1 hour at 37°C. Finally, the membranes were incubated with BCIP/NBT (Calbiochem, Bad Soden, Germany) in the dark after an incubation with streptavidin-alkaline phosphatase (R&D Systems, Minneapolis, MN) for 1 hour at RT and then visualized with a digital gel imaging analysis system.

##### 2.2.2.4 Casein zymography

The supernatant of coronary artery thrombus tissues was obtained by the methods mentioned in Western blotting. The protein (18 μL) from the tissue samples, which was added 3.6 μL 5 × SDS–PAGE loading buffer, was separated on 12% gels containing 1 mg/mL casein in separating gel. After electrophoresis, the gel was placed in an eluent (2.5% Triton X-100) eluting 2 times with shaking for 15 minutes each. The gel was immersed in the incubation medium (50 mM Tris–HCl pH 7.5, 5 mM CaCl_2_) for 16 hours at 37°C. After stained with staining solution (0.05% Coomassie Brilliant Blue, 30% methanol, 10% acetic acid) for 20 minutes, the gel was immersed in destaining solution (10% acetic acid, 5% ethanol) until the emergence of transparent strips on a blue background. Caseinolytic activity was detected as white lysis zones against a blue background.

#### 2.2.3 Plasma MMP12 and TIMP1 levels

##### 2.2.3.1 Enzyme-linked immune sorbent assay

The plasma MMP12 concentration was measured using a commercially available ELISA kit (Wuhan EIAab Science Co, Ltd, Wuhan, China). Standard, supernatant, and plasma samples were added to independent wells of microplates precoated with MMP12 specific antibodies according to the manufacturer's protocol and the analysis of preexperiment. After incubating for 90 minutes at 37°C and rinsing, 100 μL/well of biotin-conjugated antihuman MMP12 antibody (1:100 dilution) was added to the plates, except for the blank wells, incubated at 37°C for 1 hour and washed repeatedly. Afterwards, 100 μL/well of avidin–peroxidase complex (1:100 dilution) was transferred to the assay plates, except for the blank wells. After incubating at 37°C for 30 minutes in the dark and washed repeatedly, 90 μL/well tetramethyl benzidine substrate were added to each channel and incubated for 30 minutes at 37°C in the dark. Finally, the reaction was stopped by adding 100 μL stop solution. The optical density was immediately measured at 450 nm using a microplate reader.

##### 2.2.3.2 MMP12 FRET activity assay

To evaluate the activity of MMP12 in plasma, a peptide-based fluorescence resonance energy transfer (FRET) assay (SensoLyte MMP-12 Assay Kit, AnaSpec, Inc, Fremont, CA) was used and a fluorescence analyzer (Fluoroskan Ascent 2.6, Thermo) observed fluorescence intensity at excitation/emission wavelengths of 340 nm/490 nm. In brief, the blood samples (5 mL, no anticoagulant added) were centrifuged at 3000 rpm for 15 minutes at 4°C and the serum collected. Samples were incubated with APMA (4-aminophenyl mercuric acetate, in component C, AnaSpec) at a final concentration of 1 mM in the assay buffer for 2 hours at 37°C to activate MMP12 before the experiment. After activating MMP12 with APMA for 2 hours, the samples were added to a black 96-well microplate in which MMP12 substrate and an assay buffer (1:100 dilution) were mixed. Fluorescence intensities were recorded using a Fluoroskan Ascent fluorimeter at an interval of 5 minutes over a period of 1 hour until peak fluorescence was achieved.

### 2.3 Statistical analysis

On the basis of preliminary test data, a 4 ng/mL concentration difference and 3 ng/mL SD for MMP12 between STEMI and control patients was hypothesized. Considering equal number of cases and control and 80% power, the sample size was 9 patients per group. Considering a possible 10% drop-out rate, a sample size of 20 patients per group was estimated to be required. Results are expressed as means ± SEM. Skewness data were analyzed by the Kruskal–Wallis nonparametric test followed by the Mann–Whitney test pairwise comparison. Normality data were analyzed by 1-way ANOVA. In select cases, data were analyzed using the *χ*^2^ test. The correlation between different parameters was assessed by Spearman test. A *P*-value of .05 was considered statistically significant. For these analyses, SPSS v.13 (SPSS, Inc., Chicago, IL) was used. All analyses were performed using Origin 9.0 for Windows software (OriginLab Corporation, Northampton, UK, www.originlab.com).

## 3 Results

### 3.1 Clinical characteristics of the study groups

Tables 1 and 2 show the clinical characteristics of the study population. No significant differences were observed in age or sex distribution, the prevalence of major risk factors, and the level of lipid and lipoprotein among 3 groups. Inflammatory cells were significantly increased in STEMI group, which was consistent with recent knowledge that inflammation promotes atheroma formation and thrombosis.

**Table 1:**
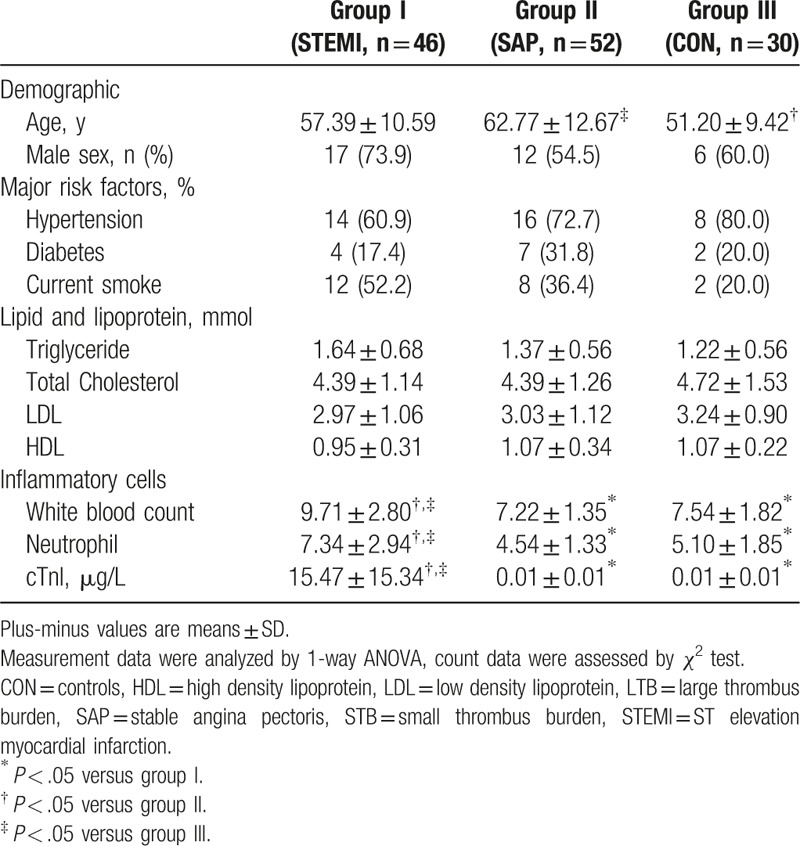
Clinical characteristics in the 3 groups of patients.

**Table 2:**
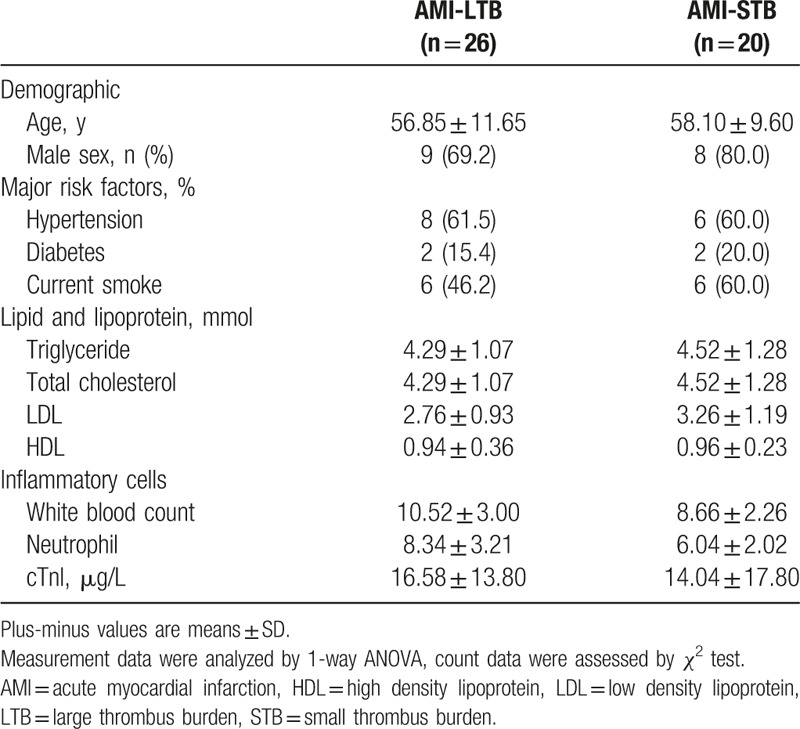
Clinical characteristics between LTB and STB groups.

In STEMI groups, baseline clinical characteristics were similar for STEMI-LTB and STEMI-STB. No complications were encountered during primary PCI. Coronary angiographies show no significant residual stenosis after stenting.

### 3.2 MMP12 is expressed on coronary thrombi

**Figure 1:**
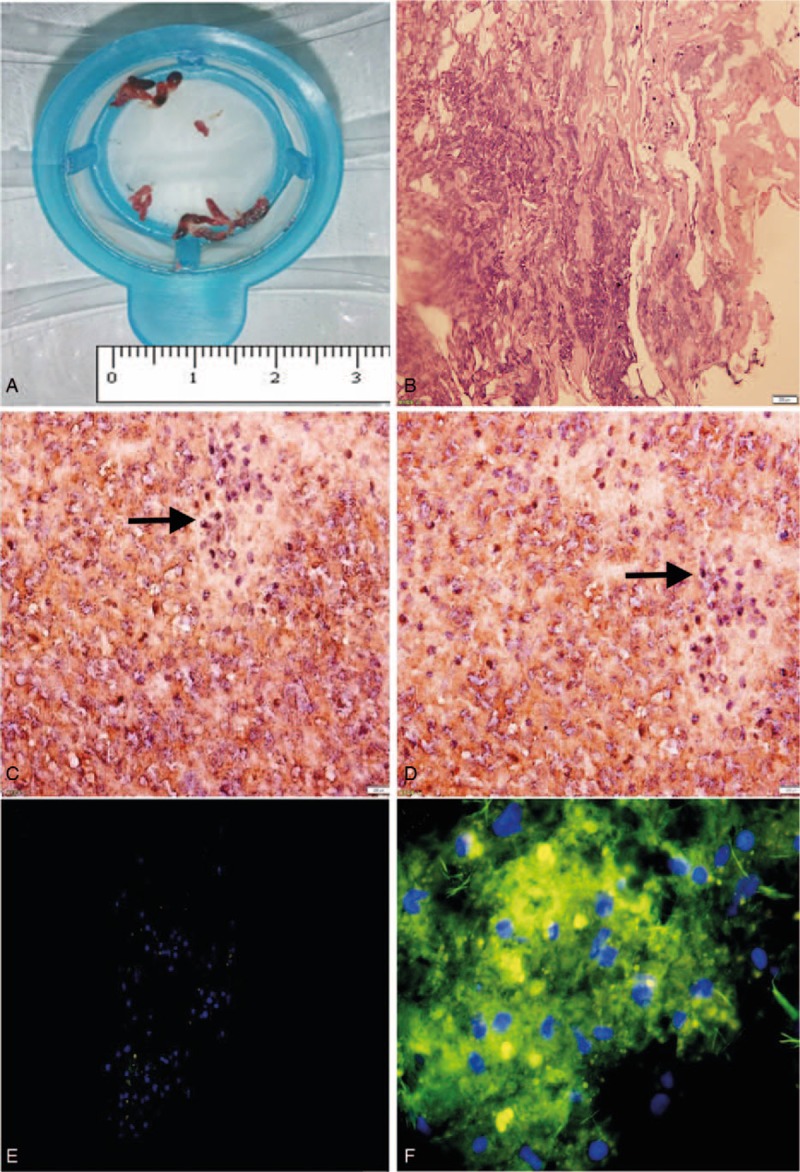
Immunohistochemistry and immunofluorescence of coronary thrombus. (A) Aspirated material in a patient with STEMI due to total occlusion of coronary artery. (B) H&E staining of coronary artery thrombus. (C and D) Immunohistochemistry of coronary thrombus with MMP12 antibody. Arrows indicate examples of MMP12 positive cells. (E and F) Immunofluorescence staining of coronary thrombus with MMP12 antibody. Chartreuse: MMP12, Blue: Counterstain nucleus with DAPI. (E) Negative control. (F) Experimental group (magnification 400×). Analysis of staining from 3 independent experiments showed similar results. STEMI = ST-segment elevation myocardial infarction.

For immunohistochemistry, coronary artery thrombus tissue specimens were obtained from a patient of STEMI-LTB. H&E staining of these specimens revealed a loose and disordered hierarchical structure with peripheral edema, mucus coagulation necrosis and an influx of inflammatory cells and red blood cells (Fig. 1B). Representative micrographs of MMP12 immunoassayed specimens are shown in Fig. 1C and D. For immunofluorescence, MMP12 was identified by immunofluorescence in coronary artery thrombus tissue specimens (Fig. 1E and F). In the control group for immunofluorescence, the thrombus was examined using 1 × PBS instead of the primary antibody and the remaining steps are the same as the experimental group. Chartreuse fluorescence of the control group was very weak, however it was bright in the experimental group.

**Figure 2:**
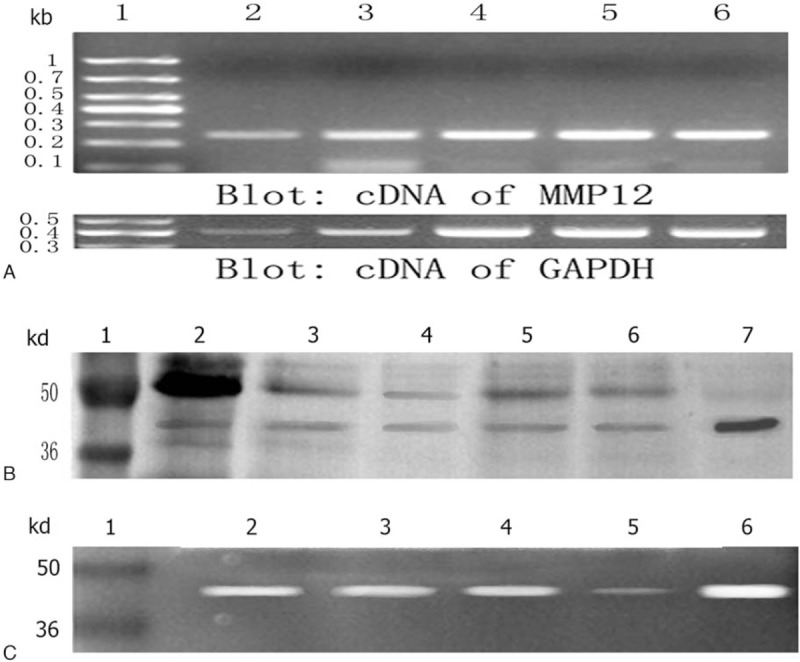
MMP12 was present in human coronary thrombi. (A) RT-PCR analysis of MMP12 gene fragment. Lane 1: DNA ladder; lanes 2–6: coronary thrombus; GAPDH blot (bottom panel) was included as loading control. (B) WB analysis of MMP12 expression in coronary artery thrombus by SDS–PAGE. Lane 1: molecular weight markers; lanes 2–6: WB products from coronary artery thrombus of STEMI-LTB; lane 7: recombinant human MMP12 with molecular weights of 45 kDa. (C) The caseinolytic activity of MMP12 in coronary thrombi. Lane 1: molecular weight markers; lanes 2–4: zymography products from the coronary artery thrombus of STEMI patients; lane 5: zymography products from the coronary artery thrombus of STEMI patients incubated with antihuman MMP12 antibody. Lane 6: Recombinant human MMP12 with molecular weights of 45 kDa. Analysis of the blots from 3 independent experiments showed similar results. LTB = large thrombus burden, RT-PCR = reverse transcription polymerase chain reaction, STEMI = ST-segment elevation myocardial infarction, WB = Western blot.

The PCR products were confirmed by nucleotide sequence analysis, which showed a 250-bp segment of the MMP12 gene (Fig. 2A). For WB, recombinant MMP12 with molecular weight of 45 kDa was identified by WB and MMP12 could be seen clearly in all cases in the thrombus groups based on calibrated molecular weight markers and recombinant MMP12 (Fig. 2B). As shown in Fig. 2C, the caseinolytic activity of MMP12 with molecular weight of 45 kDa was shown in coronary thrombus (Fig. 2C, lanes 2–4), which is in line with the result of WB. After incubation with rabbit antihuman MMP12 antibody, caseinolytic activity caused was significant attenuation (Fig. 2C, lane 5).

### 3.3 The plasma levels of MMP12 and TIMP1

#### 3.3.1 STEMI versus SAP or control group

**Figure 3:**
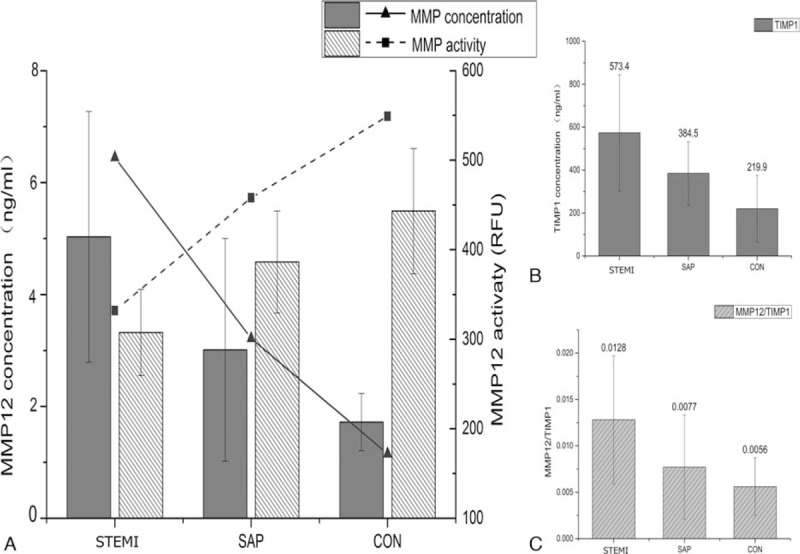
The plasma levels of MMP12 and TIMP1 in STEMI, SAP, and control groups. (A) Columns and lines show significant increase in MMP12 concentration but lower activity in STEMI compared with SAP group and normal coronary artery control group. (B) The STEMI group had significantly higher TIMP1 concentration than SAP group and control group. (C) The imbalance in MMP/TIMP ratio was observed in STEMI group compared with SAP control group. The assays were performed in triplicate and the results expressed as the mean plus or minus SEM. ^∗^*P* < .05, means significant difference between 2 groups by Mann–Whitney test. SAP = stable angina pectoris, STEMI = ST-segment elevation myocardial infarction.

MMP12 concentration measured by ELISA and activity measured by FRET were used to assess the levels in plasma from 46 STEMI, 52 SAP, and 30 control coronary artery patients. In plasma samples of the STEMI group, higher MMP12 concentration (5.030 ± 2.24 pg/mL vs 3.010 ± 1.99 pg/mL, *P* < .05) was found compared with SAP group. An even higher difference was seen between plasma samples of the STEMI group and the control group, with significantly higher MMP12 concentration (5.030 ± 2.24 pg/mL vs 1.720 ± 0.51 pg/mL, *P* < .05, Fig. 3A). A significant correlation was evident between plasma level of MMP12 and STEMI (*r* = 0.744, *P* < .0001) by Spearman test. Logistic regression analyses were calculated considering the STEMI or no-STEMI as a dependent and concentration of MMP12 as a covariate [OR = 5.459, (2.159–13.8), *P* = .000335].

However, MMP12 activity was lower in plasma samples of the STEMI group (332 ± 77 RFU) compared with SAP group (458 ± 91 RFU) and control group (549 ± 112 RFU, *P* < .05, Fig. 3A). As higher MMP12 concentrations but lower activity, we further measured the concentration of TIMP1, one of natural inhibitors of the matrix metalloproteinases, in plasma from 3 groups. In plasma samples of the STEMI group, TIMP1 concentrations were found to be higher than the SAP group (573.40 ± 270.60 pg/mL vs 384.50 ± 147.70 pg/mL, *P* < .05). Also, the STEMI group demonstrated much higher TIMP1 concentrations than the control group (573.40 ± 270.60 pg/mL vs 219.90 ± 154.80 pg/mL, *P* < .05, Fig. 3B). The imbalance in MMP/TIMP ratio was observed in STEMI group compared with SAP and control group (0.0128 ± 0.0069 vs 0.0077 ± 0.00562 vs 0.0056 ± 0.00311, *P* < .05, Fig. 3C).

#### 3.3.2 LTB versus STB subgroup

**Figure 4:**
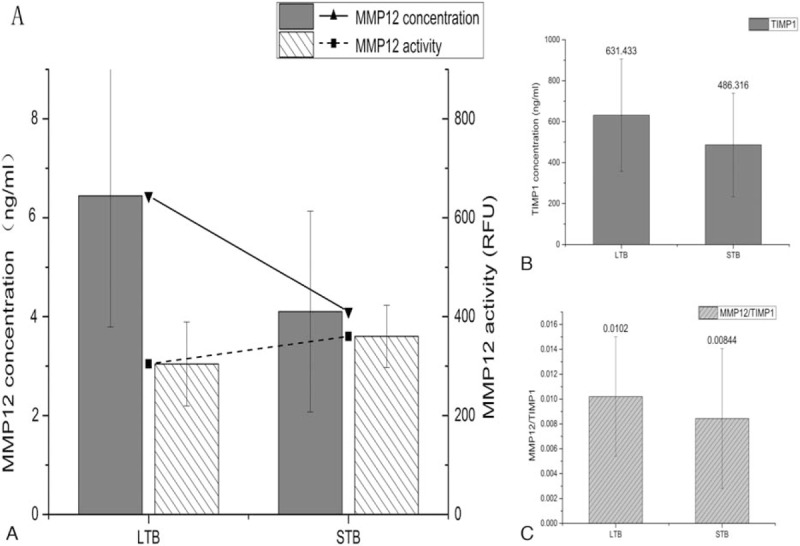
The plasma levels of MMP12 and TIMP1 in LTB and STB subgroups. (A) Columns and lines show significant increase in MMP12 concentration (*P* < .05) but lower activity (*P* > .05) in LTB compared with STB subgroup. (B) The LTB subgroup had significantly higher TIMP1 concentration than STB subgroup. (C) Although the difference did not reach statistical significance, the imbalance in MMP/TIMP ratio were higher in LTB than patients with STB. The assays were performed in triplicate and the results expressed as the mean plus or minus SEM. ^∗^*P* < .05, means significant difference between 2 groups by Mann–Whitney test. LTB = large thrombus burden, STB = small thrombus burden.

The level of MMP12 and TIMP1 measured in plasma samples of LTB and STB subgroups have similar characteristics. The higher MMP12 concentration (6.443 ± 2.652 pg/mL vs 4.103 ± 2.031 pg/mL, *P* < .05, Fig. 4A) but lower activity (304.43 ± 84.88 RFU vs 360.06 ± 63.08 RFU, *P* > .05, Fig. 4A) and higher TIMP1 concentration (631.433 ± 274.726 pg/mL vs 486.316 ± 252.498 pg/mL, *P* < .05, Fig. 4B) in LTB compared with STB was observed in this study. In addition, although the difference did not reach statistical significance, the imbalance in MMP/TIMP ratio were higher than patients with STB (0.0102 ± 0.006 vs 0.008437 ± 0.00562, *P* > .05, Fig. 4C).

## 4 Discussion

Thrombi are very frequently found in STEMI patients undergoing PCI. Intracoronary TB and associated factors are well-known contributors to risk for no-reflow, distal embolization, stent thrombosis, and long-term MACCEs.^[3]^ High thrombosis in patients has become the primary challenge to effective myocardial ischemia reperfusion, which has a risk factor on the prognosis of STEMI patients. Inflammation and increased oxidative stress play vital roles in plaque instability.^[14]^

The high expression of MMPs in the margin and shoulder of plaques induced by coronary atherosclerosis can degrade more extracellular substrate than is being synthesized, thus leading to rupture of the fiber cap. MMP1 is able to cleave and activate the thrombin receptor protease activated receptor-1 (PAR-1), which is expressed on nearly all cells in the blood, leading to signaling in the platelets in a form distinct from thrombin induced signaling.^[15]^ MMP13 can cleave and activate PAR-1, resulting in pathologic activation of downstream signaling events that contribute to platelet thrombosis and heart failure.^[16]^ Recent studies have demonstrated that MMP2 regulates the shedding of CD40 ligand from the surface of activated human platelets, suggesting MMP2 plays an important role in thrombosis.^[17]^

Although MMP12 is well investigated in emphysema, its expression and function in human coronary thrombus have never been reported.^[18]^ To our knowledge, this study provides the first report of MMP12 in human coronary thrombi (Figs. 1 and 2). We confirmed that MMP12 existed in human thrombi both at RNA and protein levels as detected by several measurements. In addition, we found elevated plasma levels of MMP12 and the imbalance of MMP12/TIMP1 in patients with STEMI compared with SAP and angiographically normal coronary control groups.

Furthermore, MMP12 concentration measured by ELISAs and activity measured by FRET were used to assess the levels in plasma from STEMI, SAP, and control patients. Interestingly, higher MMP12 concentration but lower activity was found in plasma samples of the STEMI group (Fig. 3A). Thus, we measured the concentration of TIMP1, one of natural inhibitors of the matrix metalloproteinases, in plasma from 3 groups. TIMPs are the natural inhibitor of MMP12 has been proven to have an important effect on the regulation of the MMPs function. TIMPs have 4 subtypes, TIMP1, 2, 3, 4, and TIMP1 has the highest inhibitory impact on the most active MMPs including MMP12. The expression level of TIMP1 in the plasma of the STEMI group was notably higher than the control group by ELISA and has a negative correlation with the activity of MMP12 (Fig. 3B). In addition, the ratio between MMP12 and TIMP1 was higher (Fig. 3C) in the STEMI group than other groups suggesting the increased level of MMP12 and the imbalance between MMP12 and TIMP1 in the plasma may have an important role in the incidence of STEMI. In the LTB subgroup, the higher MMP12 concentration but lower activity and higher TIMP1 concentration compared with STB was also observed. Although the difference did not reach statistical significance, the imbalance in MMP/TIMP ratio were higher in the LTB subgroup than patients with STB (Fig. 4). Thus, MMP12 and TIMP1 may have been related to TB.

Our results are in line with previous findings demonstrating that MMP12 transcript levels are significantly higher in ruptured carotid atherosclerotic plaques compared to lesions without cap disruption.^[19]^ In addition, MMP12 mRNA and protein levels markedly increase as lesions appeared and progressed in human aortic atherosclerosis.^[20]^ In a previous study of ours, we demonstrated that the proteolytic activity of MMP12 occurs within aorta and blood samples from patients with aortic dissection.^[13]^

### 4.1 Limitation

In our study, MMP12 concentrations were only measured in the peripheral plasma. In theory, transcoronary gradients of MMPs occurring between the coronary sinus (Cs) and the aorta (Ao) may more accurately reflect intracoronary release of MMPs from ruptured plaques.^[21]^ However, for patients with STEMI, the total ischemic time between the onset of symptoms and initiation of reperfusion therapy is likely the most important issue when managing STEMI.^[22]^ Guidelines for management of STEMI patients suggest that revascularization should be performed as early as possible and the first medical contact to balloon time should be less than 120 minutes.^[23]^ It certainly requires additional operational time for a multipurpose catheter to be positioned into the Cs and to collect a blood sample. As a consequence, understanding the relationship between the level of MMP12 in the peripheral plasma and TB in culprit vessel needs to be carefully assessed.

Secondly, pharmacotherapy may have influenced the release of the mediators studied. The most intensive antiplatelet treatment, as well as the statins and nitrates (widely used in acute coronary syndrome patients), are known to inhibit the expression of MMPs.^[24]^ Thus, bias introduced by pharmacotherapy needs to be noticed.

In conclusion, our case–control study results suggest that MMP12 exists in human coronary thrombus. Patients with STEMI have elevated plasma level of MMP12 and the imbalance of MMP12/TIMP1. These data supported that MMP12 might be of potential relevance in STEMI. Targeting this pathway with therapeutics is a promising strategy for treatment of patients with STEMI.

## Acknowledgment

Thanks to Mark E. Robnett and Bryan Ferguson for the English language editing.

## References

[1] Writing Group M Mozaffarian D Benjamin EJ Heart disease and stroke statistics-2016 update: a report from the American Heart Association. Circulation 2016;133:e38–60. 26673558 10.1161/CIR.0000000000000350

[2] Otsuka F Joner M Prati F Clinical classification of plaque morphology in coronary disease. Nat Rev Cardiol 2014;11:379–89. 24776706 10.1038/nrcardio.2014.62

[3] Fokkema ML Vlaar PJ Svilaas T Incidence and clinical consequences of distal embolization on the coronary angiogram after percutaneous coronary intervention for ST-elevation myocardial infarction. Eur Heart J 2009;30:908–15. 19224928 10.1093/eurheartj/ehp033

[4] Vecchio S Varani E Chechi T Coronary thrombus in patients undergoing primary PCI for STEMI: prognostic significance and management. World J Cardiol 2014;6:381–92. 24976910 10.4330/wjc.v6.i6.381 PMC4072828

[5] Davi G Patrono C Platelet activation and atherothrombosis. N Engl J Med 2007;357:2482–94. 18077812 10.1056/NEJMra071014

[6] Parks WC Wilson CL Lopez-Boado YS Matrix metalloproteinases as modulators of inflammation and innate immunity. Nat Rev Immunol 2004;4:617–29. 15286728 10.1038/nri1418

[7] Li X de Boer OJ Ploegmaker H Granulocytes in coronary thrombus evolution after myocardial infarction—time-dependent changes in expression of matrix metalloproteinases. Cardiovasc Pathol 2016;25:40–6. 26490693 10.1016/j.carpath.2015.09.007

[8] Houghton AM Quintero PA Perkins DL Elastin fragments drive disease progression in a murine model of emphysema. J Clin Invest 2006;116:753–9. 16470245 10.1172/JCI25617 PMC1361346

[9] Wang J Ye Y Wei G Matrix metalloproteinase12 facilitated platelet activation by shedding carcinoembryonic antigen related cell adhesion molecule1. Biochem Biophys Res Commun 2017;486:1103–9. 28385529 10.1016/j.bbrc.2017.04.001

[10] Gibson CM de Lemos JA Murphy SA Combination therapy with abciximab reduces angiographically evident thrombus in acute myocardial infarction: a TIMI 14 substudy. Circulation 2001;103:2550–4. 11382722 10.1161/01.cir.103.21.2550

[11] Lagerqvist B Frobert O Olivecrona GK Outcomes 1 year after thrombus aspiration for myocardial infarction. N Engl J Med 2014;371:1111–20. 25176395 10.1056/NEJMoa1405707

[12] Jolly SS Cairns JA Yusuf S Outcomes after thrombus aspiration for ST elevation myocardial infarction: 1-year follow-up of the prospective randomised TOTAL trial. Lancet 2016;387:127–35. 26474811 10.1016/S0140-6736(15)00448-1 PMC5007127

[13] Song Y Xie Y Liu F Expression of matrix metalloproteinase-12 in aortic dissection. BMC Cardiovasc Disord 2013;13:34. 23642232 10.1186/1471-2261-13-34 PMC3660235

[14] Libby P Ridker PM Hansson GK Progress and challenges in translating the biology of atherosclerosis. Nature 2011;473:317–25. 21593864 10.1038/nature10146

[15] Austin KM Covic L Kuliopulos A Matrix metalloproteases and PAR1 activation. Blood 2013;121:431–9. 23086754 10.1182/blood-2012-09-355958 PMC3548166

[16] Jaffre F Friedman AE Hu Z beta-adrenergic receptor stimulation transactivates protease-activated receptor 1 via matrix metalloproteinase 13 in cardiac cells. Circulation 2012;125:2993–3003. 22610965 10.1161/CIRCULATIONAHA.111.066787 PMC3386307

[17] Choi WS Jeon OH Kim DS CD40 ligand shedding is regulated by interaction between matrix metalloproteinase-2 and platelet integrin alpha(IIb)beta(3). J Thromb Haemost 2010;8:1364–71. 20230421 10.1111/j.1538-7836.2010.03837.x

[18] Hunninghake GM Cho MH Tesfaigzi Y MMP12, lung function, and COPD in high-risk populations. N Engl J Med 2009;361:2599–608. 20018959 10.1056/NEJMoa0904006 PMC2904064

[19] Morgan AR Rerkasem K Gallagher PJ Differences in matrix metalloproteinase-1 and matrix metalloproteinase-12 transcript levels among carotid atherosclerotic plaques with different histopathological characteristics. Stroke 2004;35:1310–5. 15073384 10.1161/01.STR.0000126822.01756.99

[20] Yu Y Koike T Kitajima S Temporal and quantitative analysis of expression of metalloproteinases (MMPs) and their endogenous inhibitors in atherosclerotic lesions. Histol Histopathol 2008;23:1503–16. 18830936 10.14670/HH-23.1503

[21] Gresele P Falcinelli E Loffredo F Platelets release matrix metalloproteinase-2 in the coronary circulation of patients with acute coronary syndromes: possible role in sustained platelet activation. Eur Heart J 2011;32:316–25. 21036774 10.1093/eurheartj/ehq390

[22] Windecker S Kolh P Authors/Task Force members. 2014 ESC/EACTS Guidelines on myocardial revascularization: The Task Force on Myocardial Revascularization of the European Society of Cardiology (ESC) and the European Association for Cardio-Thoracic Surgery (EACTS) Developed with the special contribution of the European Association of Percutaneous Cardiovascular Interventions (EAPCI). Eur Heart J 2014;35:2541–619. 25173339 10.1093/eurheartj/ehu278

[23] Steg PG James SK Task Force on the management of ST-segment elevation acute myocardial infarction of the European Society of Cardiology (ESC). ESC Guidelines for the management of acute myocardial infarction in patients presenting with ST-segment elevation. Eur Heart J 2012;33:2569–619. 22922416 10.1093/eurheartj/ehs215

[24] Fujiwara T Saito S Osanai T Decreased plasma and cardiac matrix metalloproteinase activities in patients with coronary artery disease and treated with pravastatin. Eur J Pharmacol 2008;594:146–51. 18703045 10.1016/j.ejphar.2008.07.039

